# Inuit HLA variation and neuroimmune disease

**DOI:** 10.3389/fnins.2026.1873345

**Published:** 2026-09-01

**Authors:** Angela Moreno, Candice Brinkmeyer-Langford

**Affiliations:** Department of Environmental and Occupational Health, Texas A&M School of Public Health, Texas A&M Health Science Center, College Station, TX, United States

**Keywords:** adaptation, diversity, HLA, Indigenous, neuroimmune

## Abstract

Worldwide risk of autoimmune diseases, particularly multiple sclerosis (MS), varies between populations. One key factor is immune variation attributed to genetic variation. The human leukocyte antigen (HLA) system plays a central role in antigen presentation, immune regulation, and host-pathogen interactions. Variation across HLA genes has substantial clinical implications, influencing risk for autoimmune and neurodegenerative diseases. For instance, HLA-DRB1^*^15:01 remains the strongest and most consistently replicated genetic risk factor for MS, with similar HLA-associated patterns observed in rheumatoid arthritis (RA) and systemic lupus erythematosus (SLE). Since HLA loci evolve under strong selective pressures, population-level HLA diversity reflects distinct histories of pathogen exposure, geography, climate, diet, and demographic isolation. Indigenous groups such as the First Nations, Métis, and Inuit therefore exhibit unique HLA distributions shaped by their ancestral environments. The variation in HLA genes as well as environmental factors have shaped the difference in MS prevalence and severity in these Indigenous populations compared to non-Indigenous groups.

## Introduction

Immunogenetic differences between populations shape the diversity of human leukocyte antigen (HLA) profiles and contribute to variation in disease protection and susceptibility. The HLA system is one of the most polymorphic regions of the human genome and plays a central role in antigen presentation, immune regulation, and host–pathogen interactions. Variation across HLA genes has substantial clinical implications, influencing risk for autoimmune and neurodegenerative diseases such as multiple sclerosis (MS), rheumatoid arthritis (RA), and systemic lupus erythematosus (SLE) (Trowsdale and Knight, 2013; International Multiple Sclerosis Genetics Consortium, 2011, 2019). Importantly, HLA loci evolve under strong selective pressures, and population-level HLA diversity reflects distinct histories of pathogen exposure, geography, climate, diet, and demographic isolation. Indigenous groups such as the First Nations, Métis, and Inuit therefore exhibit unique HLA distributions shaped by their ancestral environments.

Insights from studies in the Māori population of New Zealand provide a valuable context for understanding why population-specific immunogenetic analysis is essential (Tracey and Carter, 2006). The Māori, as an isolated Indigenous group, were found to have unique class II HLA allele frequencies and haplotypes distinct from those of European populations. These findings suggest that founder effects and demographic history may contribute to population-specific HLA architectures. These differences may have resulted from historical isolation, founder effects, and adaptation to local conditions, potentially leading to both the loss of certain risk alleles and the enrichment of unique, population-specific variants. Importantly, this genetic architecture could have influenced disease susceptibility and immune responses within the Māori community. However, the study did not investigate disease susceptibility or mechanisms of neuroimmune disease and therefore serves as comparative immunogenetic context rather than direct evidence for the Inuit hypothesis. The immunogenetic landscape of the Inuit has likely been shaped by isolation and local adaptation, which may have affected their disease risk similar to the Māori. The interplay of ancestry, historical demography, and selection may create distinctive immune protection and vulnerabilities, highlighting why targeted research into the HLA systems of Inuit and other Indigenous groups is crucial.

The First Nations refers to North American Indigenous populations with distinct ancestry, culture, and genetics influencing disease risk (El-Gabalawy, 2024), while the Métis represent a population with mixed European and First Nations ancestry and a correspondingly unique immunogenetic profile (Nathoo et al., 2025). The Inuit, whose ancestors adapted to Arctic environments over centuries of relative isolation, show particularly distinctive HLA patterns (Nielsen et al., 2025a).

## Literature search strategy

This Mini Review was conducted as a narrative review. Literature was identified through searches of PubMed, Google Scholar, and Web of Science using combinations of the terms “Inuit,” “Greenlandic,” “HLA,” “human leukocyte antigen,” “multiple sclerosis,” “neuroimmune disease,” “autoimmunity,” “Indigenous populations,” “immune genetics,” and “Arctic adaptation.” Priority was given to peer-reviewed studies examining Inuit populations, HLA variation, neuroimmune disease epidemiology, and immune-related genetic adaptations. Additional studies from other Indigenous populations were included when they provided relevant context regarding population-specific HLA architecture and disease susceptibility.

## The HLA system and neuroimmune risk

The HLA system governs antigen presentation to T cells, and class II alleles are particularly important in shaping immune responses to pathogens and self-antigens. While diversity in this system enhances immunoprotection, it can also predispose to autoimmune activation (Stinson et al., 2025). Recent systematic reviews highlight that specific HLA alleles are implicated in autoimmune diseases such as type 1 diabetes, rheumatoid arthritis, and MS, in which antigen presentation drives inappropriate immune responses (Berryman et al., 2023). HLA variation can also interact with the gut immune axis, influencing comorbidities and modulating disease severity (Berryman et al., 2023).

Within this framework, the distinctive HLA distributions observed in the Inuit, shaped by long-term Arctic adaptation, historical isolation, and unique pathogen exposures, may contribute to the relatively low prevalence of certain autoimmune and neuroimmune diseases observed in Inuit populations. Direct evidence from Inuit populations demonstrates an unusually low prevalence of MS, together with distinctive HLA allele frequencies and immune-related genetic variation. However, direct genotype–phenotype studies linking these variants to neuroimmune disease susceptibility remain unavailable. Therefore, whether Inuit-specific HLA profiles directly influence disease severity following disease onset remains unclear and requires further investigation. Although Indigenous groups such as the First Nations, Métis, and Inuit all possess unique immunogenetic profiles, the Inuit represent a particularly compelling case for examining how population-specific HLA variation may be a factor in suppressing disease onset and intensifying downstream neurodegenerative processes. Inuit-specific immunogenetic architecture may influence both protection and vulnerability. For example, although the Inuit exhibit a markedly lower prevalence of MS than non-Indigenous groups, Inuit individuals who do develop MS experience significantly more severe disease, i.e., faster disability progression, including more rapid attainment of an Expanded Disability Status Scale (EDSS) score exceeding 6, increased neurological disability, and greater functional impairment rather than increased disease incidence or mortality (Saeedi et al., 2012; Nathoo et al., 2025). Specifically, compared to all MS patients in British Columbia, MS patients who were members of Indigenous groups in British Columbia showed faster disease progression to an EDSS score of over 6 (Saeedi et al., 2012).

## Distinctive Inuit immunogenetics: protection and vulnerability

Emerging research further underscores the unique role of Inuit immunogenetics in shaping their risk for autoimmune and neurodegenerative diseases. Although the Inuit exhibit markedly lower rates of autoimmune disorders such as MS, their disease burden, when it occurs, is disproportionately severe (Saeedi et al., 2012; Nielsen et al., 2025a). This contrast suggests that biological, environmental, and demographic factors may contribute to differences in disease patterns observed between Inuit and non-Inuit populations.

One possible explanation is the presence of genetic variants shaped by long-term adaptation to Arctic environments, dietary patterns, and historical isolation, which influence immune function and disease expression (Stinson et al., 2025). Evidence from studies on inborn errors of immunity in Inuit children further illustrates how rare immune dysregulation can present with heightened clinical impact, highlighting the clinical impact that rare immune disorders can have within founder or geographically isolated populations (Pham-Huy et al., 2024). However, these findings do not necessarily indicate population-wide differences in immune responses among Inuit individuals.

Adaptive pressures have also shaped specific immune-related genes. Direct genetic evidence from Greenlandic Inuit comes from the proteogenomic study of Stinson et al. (2025), which identified Inuit-enriched variants associated with altered plasma concentrations of several immune-related proteins, including interleukin 27 (IL27), interleukin 16 (IL16), Galectin-9, and IgG Fc receptor IIb. Importantly, this study did not examine multiple sclerosis, HLA function, or neuroimmune disease outcomes. Therefore, these findings provide indirect evidence that immune regulatory pathways differ in Inuit populations but should not be interpreted as demonstrating altered autoimmune susceptibility or HLA-mediated neuroprotection. There is additionally a very low frequency of the major MS risk allele, HLA-DRB1^*^15:01, in Inuit populations (Metcalfe et al., 2013). The low frequency of HLA-DRB1^*^15:01 may contribute to reduced MS susceptibility among Inuit populations; however, disease risk is likely influenced by the broader structure of HLA haplotypes and additional genetic and environmental factors. Arctic-adapted variants in genes such as carnitine palmitoyl transferase 1A (CPT1A) and hepatocyte nuclear factor 1-alpha (HNF1A) influence vitamin D metabolism and lipid-immune pathways, further shaping immune responses that differ from those observed at lower latitudes (Stinson et al., 2025). Mutations in the CPT1A gene found in Inuit populations include a cytosine to thymine transition at position 1436, leading to a substitution of proline for leucine at codon 479 (Rajakumar et al., 2009; Collins et al., 2010). The CPT1A protein has a crucial function in the transfer of long-chain fatty acids to mitochondria to be used as fuel, leading to an important role in energy production. The clinical implications of this particular mutation have been characterized as reduced activity of CPT1A leading to increased glycolysis (Brown et al., 2001; Collins et al., 2010), which has been correlated with improved symptoms in the experimental autoimmune encephalitis mouse model of MS (Mørkholt et al., 2019). Furthermore, the G319S variant of HNF1A is associated with type 2 diabetes solely in Aboriginal Canadians (Ley et al., 2011), and a novel splice-site mutation in the HNF1A gene is found only in Greenland Inuit with type 2 diabetes (Thuesen et al., 2022). Although these findings suggest population-specific differences in immune regulatory pathways, direct links between these variants and autoimmune disease susceptibility have not yet been established.

**Figure 1:**
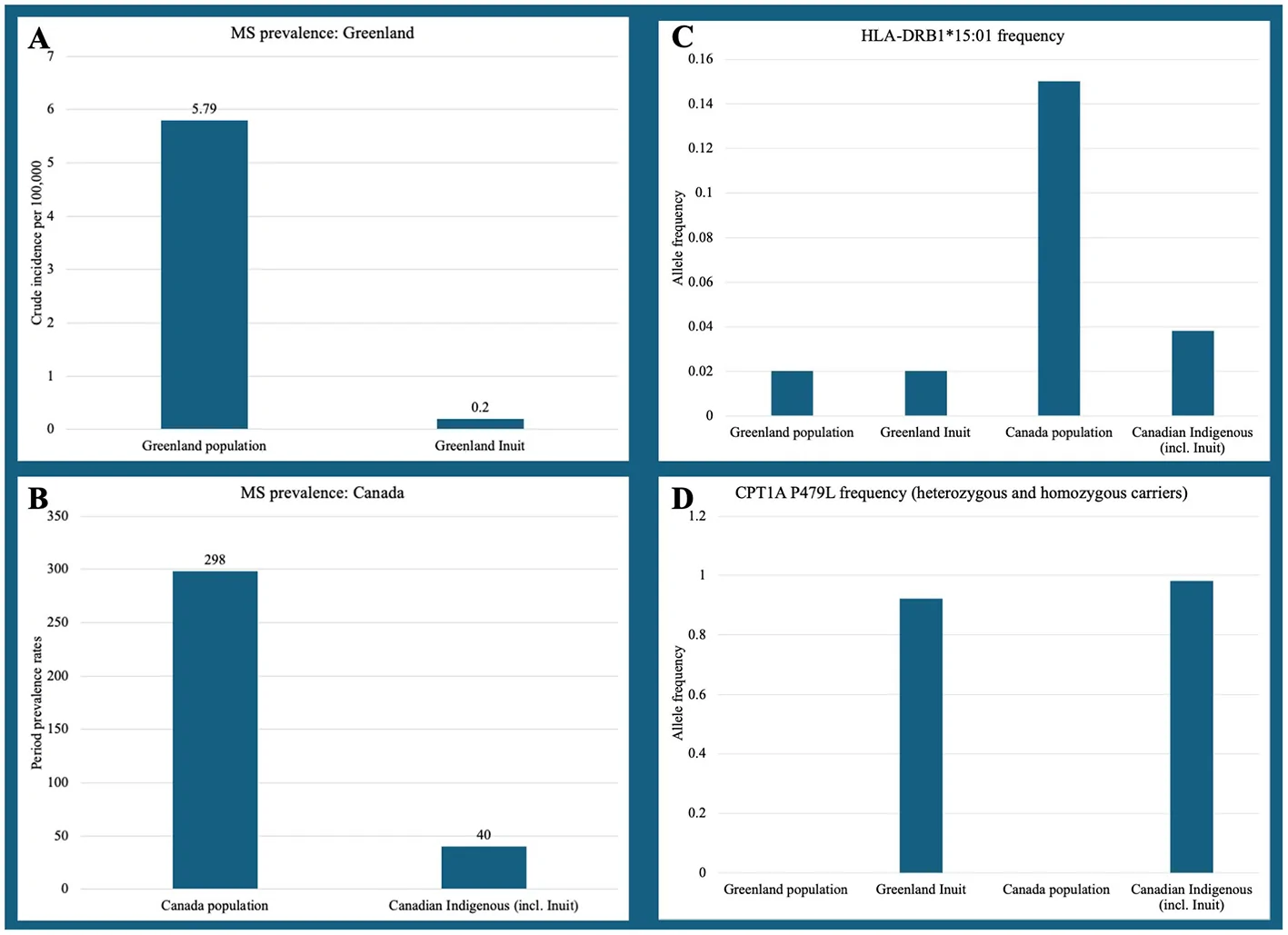
Differences in MS prevalence in Inuit vs. non-Inuit populations may correlate with different allele frequencies of key loci. **(A, B)** Show the difference in MS prevalence per 100,000 people in the Inuit and non-Inuit populations of Canada and Greenland. Sources: Crude incidence rates for Greenland and Greenland Inuit are from Nielsen et al. (2025b); period prevalence rates for Canada and Canadian Indigenous are from Mirsattari et al. (2001). **(C)** Focuses on frequency of the MS risk allele HLA-DRB1*15:01 in these populations. Sources: Metcalfe et al. (2013) and Gonzalez-Galarza et al. (2020). **(D)** Illustrates the frequency of the CPT1A P479L variant in these populations. Sources: Rajakumar et al. (2009) and Mørkholt et al. (2019).

Figure 1 summarizes the key differences related to MS risk.

## Comparison: Inuit and non-Inuit in Greenland and Denmark

Non-Inuit living in Greenland represent a distinct comparison group. Neurological disease patterns differ between Inuit residents of Greenland, non-Inuit in Greenland, and non-Inuit in Denmark. Non-Inuit residing in Greenland are found to have generally lower risks for major neurological disorders than Danes, despite living in a similar environment and having comparable access to healthcare (Nielsen et al., 2025b). Indeed, Inuit living in Greenland had a lower MS incidence rate than Inuit living in Denmark (< 0.20 vs. 2.07 per 100,000 person-years of risk, Nielsen et al., 2025b). This reflects the overall pattern of increased MS prevalence in the Danish population compared to Greenland, though for both Denmark and Greenland, Inuit populations had reduced prevalence of MS compared to that of non-Inuits. Healthier lifestyle patterns among Greenlanders, such as more favorable dietary habits, higher levels of physical activity, and lower smoking rates, may contribute to these differences. However, these environmental and lifestyle differences only partly explain the observed variation in disease risk (Nielsen et al., 2025a).

Importantly, non-Inuit in Greenland do not display the same profile of infection-related neurological risks as Inuit residents. Inuit individuals have elevated risks for epilepsy, meningitis, and other central nervous system infections, whereas non-Inuit in Greenland do not demonstrate these same increases (Nielsen et al., 2025a). This divergence suggests that the heightened vulnerability to certain infection-related neurological disorders observed in Inuit groups is not shared by non-Inuit residents, underscoring the role of population-specific biological and historical factors in disease expression (Nielsen et al., 2025a). Non-Inuit in Greenland occupy an intermediate epidemiological position, with a neurological disease burden lower than non-Inuit in Denmark but without the same infection-related risks seen in Inuit populations (Nielsen et al., 1997). These patterns highlight how ancestry and environment interact to shape neurological health, with non-Inuit in Greenland influenced primarily by environmental exposures, while the Inuit are also shaped by long-term Arctic-adapted immunogenetics. The differing rates of neurological conditions observed between Inuit and non-Inuit populations suggest that genetic factors may contribute to disease susceptibility; however, current epidemiological studies do not directly establish a protective effect of specific Inuit HLA variants.

A summary of the findings in these Indigenous populations described here is found in Table 1.

**Table 1:** Summary of studies describing aspects of risk of neuroimmune disease in different populations, specifically those factors related to genetic background.

Study	Population	Data type	Main finding	Relevance to Inuit neuroimmune risk
Nielsen et al. (2025b)	Greenlandic population/Inuit	MS epidemiology	MS prevalence remains low in Greenland; estimated 48.29/100,000 in 2014	Direct
Saeedi et al. (2012)	Aboriginal/Indigenous MS patients in British Columbia	Clinical MS cohort	Lower prevalence but more severe disease course reported among Indigenous MS patients	Partial/direct
Nathoo et al. (2025)	Indigenous Peoples with MS in Canada	Review/clinical synthesis	Indigenous people with MS may experience greater severity and distinct care gaps	Partial/direct
Metcalfe et al. (2013)	Nunavik Inuit women	HLA allele frequency	Low frequency of MS-associated HLA alleles: DRB1^*^15:01 3.8%, DQB1^*^06:02 3.63%	Direct genetic context
Stinson et al. (2025)	Greenlandic Inuit	Proteogenomics/pQTLs	Inuit-enriched immune-related variants linked to IL27, IL16, Galectin-9, Fc receptor pathways	Indirect mechanistic support
Pham-Huy et al. (2024)	Canadian First Nations and Nunavut Inuit children	Inborn errors of immunity	Rare immune dysregulation can have high clinical impact in founder/isolated populations	Indirect/contextual
Collins et al. (2010)	Yukon, Northwest Territories, Nunavut newborns	CPT1A population genetics	CPT1A P479L is common in northern Indigenous/Inuit populations	Indirect metabolic-immune context
Rajakumar et al. (2009)	Greenland Inuit/Canadian Inuit context	CPT1A genetics/metabolism	CPT1A P479L is common in Greenland Inuit and associated with lipid traits	Indirect metabolic-immune context
Mørkholt et al. (2019)	Experimental autoimmune encephalomyelitis model	Functional MS/EAE biology	CPT1A modulation influenced EAE/MS-like disease biology	Mechanistic context, not Inuit-specific
Thuesen et al. (2022)	Greenlandic Inuit	HNF1A genetics/diabetes	Novel splice-affecting HNF1A variant has large population impact on diabetes in Greenland	Indirect metabolic-immune context
Ley et al. (2011)	Aboriginal Canadians	HNF1A G319S/diabetes	HNF1A G319S associated with type 2 diabetes in Aboriginal Canadians	Context only; not Inuit-specific
Tracey and Carter (2006)	Māori	HLA class II genetics	Māori have population-specific DRB1, DQB1, and DPB1 allele/haplotype architecture	Comparative Indigenous context

## Population specific patterns in a global context

Global analyses of HLA variation further support the distinct immunogenetic patterns seen in the Inuit. Large-scale studies identify clustering of HLA-DRB1 alleles among Indigenous populations across the Americas, such as the Quechua, Aymara, and Mesoamerican groups, all of which differ markedly from Europeans (Arnaiz-Villena et al., 2020; Arrieta-Bolaños et al., 2023). These patterns reflect evolutionary histories shaped by migration, geographic isolation, and local environmental pressures. For example, HLA-DRB1 allele frequencies of populations from northeastern Asia cluster with Indigenous Alaskans and Greenland Inuit, while other Indigenous groups from the Americas cluster separately (Arrieta-Bolaños et al., 2023), likely reflecting differences in pathogen exposure, geographic isolation and migration. The relatively low frequency of MS risk alleles, including HLA-DRB1^*^15:01, is consistent across many Indigenous populations, reinforcing that neuroimmune disease risk cannot be attributed to a single allele but instead reflects the broader structure of population-specific HLA profiles.

Comparative evidence from other Indigenous populations provides additional context regarding the effects of founder events and geographic isolation on HLA architecture. The complexity of making precise mechanistic links between population-specific HLA allele frequencies, haplotype structures, and disease risk is highlighted by the Māori experience. Māori populations exhibit distinctive HLA class II haplotypes compared with Europeans, suggesting that demographic history can shape immunogenetic diversity. Founder effects and historical demographic isolation may have led to the enrichment of certain alleles in the Māori, with implications for disease risk and immune function (Tracey and Carter, 2006). Although informative, these findings should not be interpreted as direct evidence for Inuit disease mechanisms. For the Inuit, it is plausible that variants favored by Arctic adaptation alter the threshold for autoreactive T-cell priming, limiting disease initiation, but that population-enriched regulators of inflammatory balance may amplify downstream immune responses once activation begins. At present, this hypothesis has not been directly tested in Inuit populations. This possibility highlights a current gap in the literature and underscores the need for studies that connect population-genetic variation with functional immune behavior throughout disease progression. Although findings from Māori populations cannot be directly extrapolated to Inuit populations, they provide an example of how population-specific HLA architecture may influence disease susceptibility and immune responses.

## Non-genetic risk factors for multiple sclerosis

Beyond genetics, a multitude of environmental and lifestyle factors are now recognized as important contributors to the risk of multiple sclerosis. Vitamin D deficiency, a consequence of limited sun exposure or dietary insufficiency, has emerged as a significant risk, as vitamin D plays a vital regulatory role in immune function and inflammatory balance (Olsson et al., 2016). Importantly, epidemiological evidence shows that regions at higher latitudes, such as Alaska and Canada, experience reduced sunlight and ultraviolet exposure, leading to lower vitamin D production and significantly higher MS prevalence. The latitude gradient for MS risk is strongly established in early life, with prevalence increasing as one moves farther from the equator, and this effect is likely driven by environmental exposures as early as birth or even *in utero* (Sabel et al., 2021). However, traditional Inuit diets are high in fatty fish and marine mammals, which has enabled these populations to bypass the reliance on UV-induced vitamin D synthesis (Kuhnlein and Receveur, 1996). As these traditional diets are transitioned to more modern nutrition, Inuit populations have started to see a greater deficiency in vitamin D (Irvine and Ward, 2022), which may contribute to increased disease risks in the future.

Other non-genetic MS risk factors include obesity, particularly during adolescence, which is associated with increased MS risk likely through its effects on systemic inflammation and immune dysregulation (Munger et al., 2009). Inuit have different abdominal fat distribution compared to other ethnic groups (Rønn et al., 2017), with different correlations to mortality and other health factors. Smoking is a consistently reported risk factor, with evidence indicating both direct inflammatory effects and interactions with genetic predisposition (Hedström et al., 2013). Smoking is a factor for Inuit populations as well, though rates have lowered since the 1990s (Bougie and Kohen, 2017). Viral exposures, most notably Epstein–Barr virus (EBV), have been shown to increase susceptibility; individuals who have experienced infectious mononucleosis demonstrate a notably higher likelihood of developing MS later in life (Handel et al., 2010; Bjornevik et al., 2022). Inuit populations, specifically in Greenland, appear to have an EBV rate comparable to that of other groups (Albeck et al., 1985), suggesting that EBV infection alone is insufficient to increase MS risk in all populations. More recently, the composition of the gut microbiome has gained attention, as imbalances in beneficial bacteria can influence immune activation and may worsen MS symptoms or progression (Cekanaviciute et al., 2017). Antibiotic use, which disrupts gut microbial ecology, is also being explored as a potential risk modifier, due to its impact on immune signaling via the gut-brain axis. Diet, as both a source of vitamin D and a determinant of microbiome structure, further shapes MS risk and course. Collectively, these factors underscore a complex interplay between environment, lifestyle, and immune regulation, highlighting that risk for MS is shaped not only by inherited immunogenetic profiles but also by the lived context of individuals and populations.

Table 2 provides a summary of population-specific features, particularly in relation to immunogenetics.

**Table 2:** Population-specific immunogenetic profiles reveal how indigenous populations are far less studied than European populations.

Population	Dominant HLA features	Other adaptive variants	MS prevalence	Proposed evolutionary pressure	Knowledge gaps
Inuit	Low DRB1^*^15:01; distinct class II profile	CPT1A (P479L), HNF1A, IL27, IL16	Low (~48/100 k) but highly severe	Arctic climate, diet, pathogen history	Mechanism of severe disease
Māori	Distinct DRB1/DQB1 haplotypes	Limited functional proteomic data	Low to moderate (partially masked by under-reporting)	Founder effects, isolation	Functional immune studies
First Nations	High HLA class II diversity; distinct from European cohorts	HNF1A G319S (specific groups)	Variable (geographic gradients observed)	Local adaptation	Limited HLA studies
Métis	Mixed Indigenous-European immunogenetic framework	Poorly characterized metabolic variants	Intermediate (reflecting mosaic population)	Admixture	Scarcity of targeted immunogenetic cohorts
Europeans	High DRB1^*^15:01 frequency (~15–30%)	Standard non-Arctic baseline variants	High (>100–200/100 k)	Different pathogen history	Over-represented baseline; lacks insights from non-temperate adaptations

Specific genetic variants, in combination with each population's environmental heritage, may contribute to overall MS prevalence.

## Gaps in mechanistic understanding

Although immunogenetic differences among Indigenous populations, including the First Nations, Métis, and Inuit, are being increasingly described, the specific mechanisms by which the distinct HLA profile of the Inuit produces low rates of autoimmune and neurodegenerative disease yet severe outcomes in cases remain unclear. While existing data document the rarity of major risk alleles in the Inuit, the presence of population-enriched variants that influence cytokine signaling, lipid-immune pathways, and other features shaped by Arctic selective pressures, there is not yet an integrated explanation of how these variants work together to modify the initiation and course of disease.

**Figure 2:**
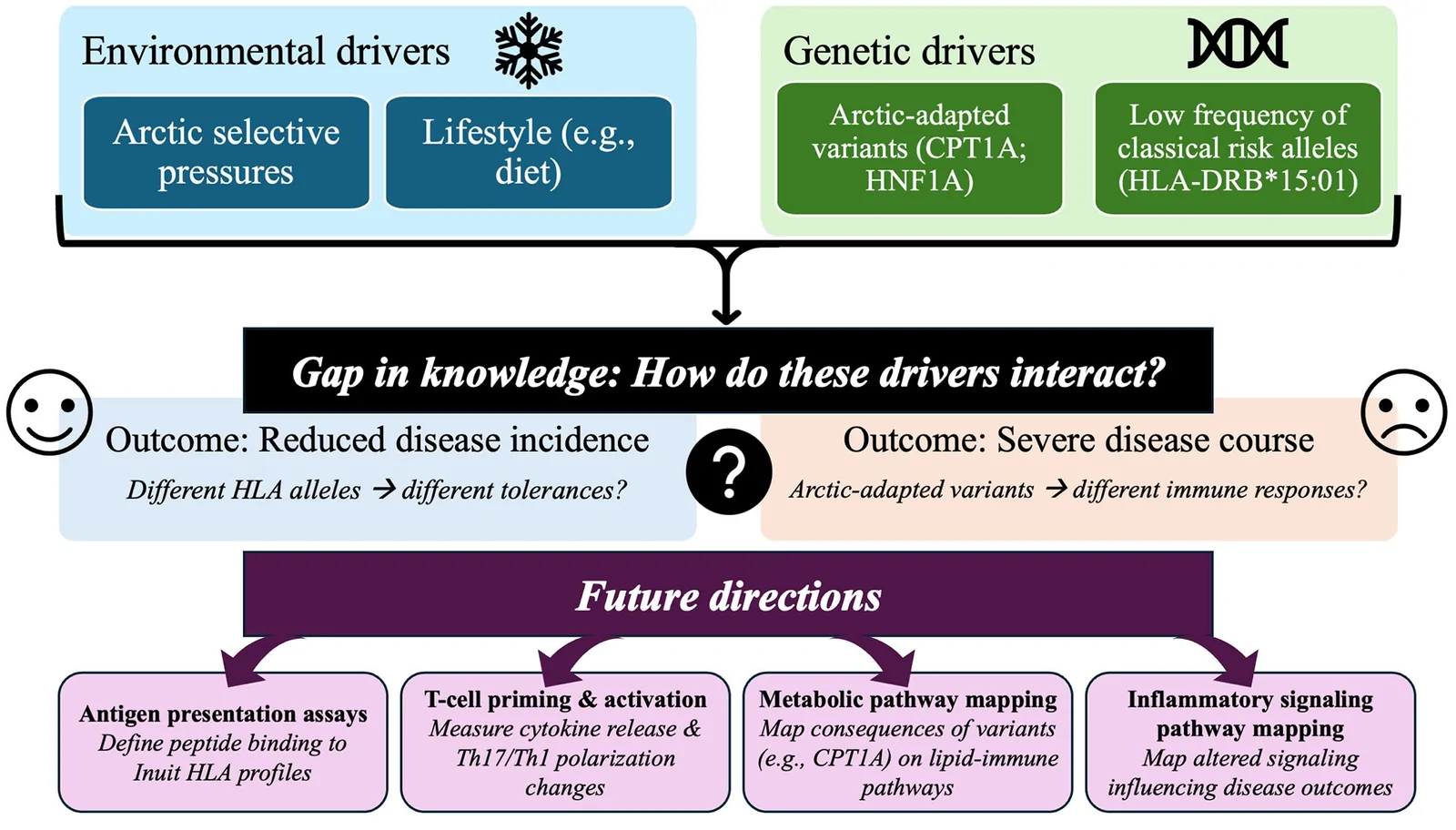
Summary of the Inuit immunogenetic paradox and potential future directions. At the top, Inuit-specific environmental and genetic factors are listed, and the middle section defines the “paradox” of the outcomes of interactions between these drivers (possible disease protection with greater disease severity in those Inuit who do develop the disease). Along the bottom are possible methods that could be used to help resolve the gap in knowledge regarding the interaction between environmental and genetic factors.

Addressing this will require mechanistic studies that bridge population-genetic data with functional analyses of antigen presentation, T-cell priming, cytokine regulation, and inflammatory signaling. The complexity of the topic is compounded by findings in population-genetic studies from other Indigenous groups in the Americas and the Pacific, which show that evolutionary processes such as migration, bottleneck effects, and adaptation have shaped patterns of HLA class II allele diversity and thereby influenced disease susceptibility (Arrieta-Bolaños et al., 2023). Figure 2 provides a summary of the gap in understanding regarding reduced disease susceptibility coupled with increased disease severity in Inuit populations.

## Discussion

The immunogenetic architecture of the Inuit, shaped by Arctic adaptation and generations of isolation, helps explain how population-specific evolutionary histories create both resilience and vulnerability in neuroimmune disease. This immunogenetic profile represents a potential paradox. Their exceptionally low prevalence of autoimmune and neurodegenerative disorders, paired with disproportionately severe outcomes when disease does arise, indicates an immune system potentially influenced by powerful selective pressures unique to the Arctic, such as arctic climate, distinct pathogen exposure, and geographic isolation. Over centuries, this may have shaped the Inuit immune system for handling acute infectious threats or unique dietary metabolic demands, potentially affecting susceptibility to autoimmunity. Furthermore, while this review primarily focuses on HLA class II because of its dominant role in CD4? T-cell activation and the well-established association of HLA-DRB1^*^15:01 with MS susceptibility, class I molecules also contribute to disease risk. Several HLA-A and HLA-B alleles have been associated with reduced MS susceptibility or modified disease progression, likely through effects on CD8? T-cell responses and immune surveillance (Fogdell-Hahn et al., 2000; Patsopoulos et al., 2013, International Multiple Sclerosis Genetics Consortium, 2019). However, relatively little is currently known regarding the distribution or functional significance of HLA class I alleles in Inuit populations, representing another important area for future investigation.

As demonstrated in the Māori and other Indigenous groups, unique HLA class II allele distributions drive population-specific patterns of disease risk and immune response (Tracey and Carter, 2006). Although the low frequency of high-risk HLA alleles combined with Inuit-enriched immune variants suggests a possible genetic contribution to the reduced disease onset, the paradoxical severity in clinical outcomes is still unexplained. While the low frequency of classical risk alleles may affect the likelihood of autoimmunity, an intense environmental trigger (like a severe CNS infection) can potentially influence the development of autoimmune responses. Once activated, the population-enriched variants in certain immune-related pathways (IL27, IL16, Galectin-9, Fc receptor pathways) may affect inflammatory responses related to autoimmune outcomes in ways that contrast to the slow, chronic smoldering seen in European cohorts.

It is increasingly clear, however, that non-genetic risk factors must also be considered to fully explain these disease patterns. For example, environmental exposures such as sustained vitamin D deficiency, linked to low sunlight at high latitudes, may modify immune function despite genetic adaptations in vitamin D metabolism. For the Inuit, the historical protection of a marine diet (abundant in preformed vitamin D, bypassing UV reliance) could be affected by modern shifts toward market foods, creating a novel systemic vulnerability (Kuhnlein and Receveur, 1996). Obesity, smoking, and viral exposures, including Epstein-Barr virus, are further established contributors to MS risk and progression (Hedström et al., 2013), potentially interacting with Inuit-specific immunogenetic backgrounds to influence disease severity. High regional smoking rates in Inuit populations (Bougie and Kohen, 2017), and rising adolescent BMI, could introduce massive pro-inflammatory variables that directly counter baseline genetic protections by aggravating blood-brain barrier permeability and triggering systemic adipokine dysregulation (Rønn et al., 2017; Lajeunesse-Trempe et al., 2024). Furthermore, while EBV is near-universal, the distinct early-childhood seroconversion pattern observed in the Arctic may fundamentally alter how memory T-cell pools are primed compared to temperate-zone populations. Moreover, recent attention to gut microbiome composition and dietary patterns highlights that lifestyle factors modulate immune activation and may exacerbate clinical outcomes in susceptible individuals.

Comparisons with non-Inuit populations in Greenland and Denmark reinforce the importance of environmental and lifestyle factors. While non-Inuit in Greenland have historically benefitted from arguably healthier lifestyle patterns and lower neurological disease risk than Danes, they do not exhibit the infection-related vulnerabilities seen in Inuit populations, showing that disease risk reflects both genetic and environmental factors. Thus, an integrated approach considering both genetic and non-genetic determinants is essential for understanding neuroimmune disease expression in Arctic populations.

Despite substantial progress in identifying potential contributors, including the low frequency of high-risk HLA alleles and the presence of Inuit-enriched variants influencing immune regulation, a coherent explanation of the discordant effects of Inuit immunogenetics remains elusive. Future studies must focus on integrating population-genetic analysis with detailed immunological and clinical investigation, ideally in longitudinal designs, to clarify how these diverse genetic and environmental factors combine to shape disease initiation, progression, and outcomes. Studying highly adapted populations like the Inuit, Māori, or First Nations demonstrates that “universal” genetic risk models (largely built on European cohorts) fail in non-temperate contexts. Such research will not only provide answers relevant to the Inuit but will also broaden our understanding of human neuroimmune diversity. Ultimately, this broadened understanding will lead to highly targeted, population-specific therapeutic interventions.
